# Risk Factors for Seabird Bycatch in a Pelagic Longline Tuna Fishery

**DOI:** 10.1371/journal.pone.0155477

**Published:** 2016-05-18

**Authors:** Eric Gilman, Milani Chaloupka, John Peschon, Sarah Ellgen

**Affiliations:** 1 Pelagic Fisheries Research Group, Honolulu, United States of America; 2 Ecological Modeling Services and University of Queensland, St. Lucia, Australia; 3 Pacific Islands Regional Office, National Marine Fisheries Service, Honolulu, United States of America; Hellenic Centre for Marine Research, GREECE

## Abstract

Capture in global pelagic longline fisheries threatens the viability of some seabird populations. The Hawaii longline tuna fishery annually catches hundreds of seabirds, primarily Laysan (*Phoebastria immutabilis*) and black-footed (*P*. *nigripes*) albatrosses. Since seabird regulations were introduced in 2001, the seabird catch rate has declined 74%. However, over the past decade, seabird catch levels significantly increased due to significant increasing trends in both effort and nominal seabird catch rates. We modelled observer data using a spatio-temporal generalized additive mixed model with zero-inflated Poisson likelihood to determine the significance of the effect of various risk factors on the seabird catch rate. The seabird catch rate significantly increased as annual mean multivariate ENSO index values increased, suggesting that decreasing ocean productivity observed in recent years in the central north Pacific may have contributed to the increasing trend in nominal seabird catch rate. A significant increasing trend in number of albatrosses attending vessels, possibly linked to declining regional ocean productivity and increasing absolute abundance of black-footed albatrosses, may also have contributed to the increasing nominal seabird catch rate. Largest opportunities for reductions are through augmented efficacy of seabird bycatch mitigation north of 23° N where mitigation methods are required and during setting instead of during hauling. Both side vs. stern setting, and blue-dyed vs. untreated bait significantly reduced the seabird catch rate. Of two options for meeting regulatory requirements, side setting had a significantly lower seabird catch rate than blue-dyed bait. There was significant spatio-temporal and seasonal variation in the risk of seabird capture with highest catch rates in April and May and to the northwest of the main Hawaiian Islands.

## Introduction

Pelagic longline and other marine capture fisheries that target relatively fecund species with r-selected life history characteristics, such as tuna and tuna-like species (Scombridae), can have large impacts on incidentally caught species with K-selected life-history strategies, including seabirds, sea turtles, marine mammals, elasmobranchs and some bony fishes [1, 2]. These K-selected associated and dependent species have relatively long lifespans, slow growth, late sexual maturity, low fecundity and low natural mortality rates. As a result, they have low resistance and resilience to even low levels of anthropogenic mortality [3–6].

At least 160,000 seabirds are estimated to be killed annually in pelagic and demersal longline fisheries worldwide, threatening the viability of some populations of albatrosses, petrels, shearwaters and other species [1, 7–10]. Seabird bycatch in longline fisheries occurs primarily in higher latitudes. Seabirds are captured predominantly while fishing gear is being set: seabirds are hooked or entangled, and in fisheries where gear soaks below the sea surface, the captured birds are dragged underwater as the gear sinks [2,8].

The Hawaii deep-set longline tuna fishery lands primarily bigeye tuna (*Thunnus obesus*) and also retains several other tuna and tuna-like species (Scombroidei) and billfishes. In 2012, 128 active vessels landed about 10,400 t [11, 12]. The observer coverage rate of the Hawaii longline tuna fishery was about 4% from 1994 to 2000, after which it increased to about 20% [13]. Regulations to mitigate seabird bycatch were first adopted in 2001. The most recent amendment, which came into effect in 2006, defines two alternative suites of seabird bycatch mitigation methods. Deep-setting vessels are required to use one of these two suites of measures when fishing north of 23° N. latitude. Vessels can opt to (i) set gear from the side of the vessel; set the mainline and mount a line shooter, if used, ≥ 1 m from the stern corner; deploy a bird curtain aft of the line shooter that meets regulatory design specifications; and use a minimum of 45 g weights attached within 1 m of the hook, referred to hereafter as the suite of measures that includes side setting. Or, vessels can (ii) discharge fish, offal or spent bait, with all fish hooks removed, from the opposite side of the vessel where gear is being set or hauled when seabirds are present; use thawed and blue-dyed bait; use a mainline line shooter; and use a minimum of 45 g weights attached within 1 m of the hook, referred to hereafter as the suite of measures that includes blue-dyed bait [13, 14]. See Gilman et al. [15–17] and WPFMC [12] for more information on the Hawaii longline tuna fishery’s gear, methods and catch.

The Hawaii longline tuna fishery catches primarily Laysan (*Phoebastria immutabilis*) and black-footed (*P*. *nigripes*) albatrosses. Since observer coverage in the fishery began in 1994 through 2013, observers recorded the capture of 710 seabirds. Of these, 47% were Laysan albatrosses, 46% black-footed albatrosses, and 6% shearwater spp. (likely sooty shearwaters *Ardenna grissea* or short-tailed shearwaters *A*. *tenuirostris*),. The remainder was one observed capture each of a brown booby (*Sula leucogaster*) and red-footed booby (*S*. *sula*) and six unidentified species of seabirds [13, 18–20]. The IUCN Red List categorizes Laysan and black-footed albatrosses as Near Threatened, sooty shearwaters as Near Threatened, short-tailed shearwaters as Least Concern, and brown and red-footed boobies as Least Concern [21]. These species are not listed as endangered or threatened under the U.S. Endangered Species Act [13].

Gilman et al. [15] analyzed observer data from the Hawaii longline tuna fishery through 2007, finding a 67% significant reduction in the standardized seabird catch rate following the introduction of regulations in 2001 to manage seabird bycatch. For the most recent decade for which fleet-wide raised seabird catch levels have been estimated, based on modeling observer data from about a 20% observer coverage rate [22–31], from 2004 to 2013 there was a significant increasing trend in the estimated raised number of seabirds caught per year, increasing by about 45 seabird captures per year, based on fitting data to a linear regression model (p<0.001, R^2^ = 0.85). A mean of 210 seabirds (± 92 95% CI) were estimated to be captured by the fishery annually during this period. The estimates of annual catch levels, however, do not account for various sources of fishing mortality, such as unobserved pre-catch events, post-release mortality, ghost fishing mortality, and indirect collateral sources of mortality, which can be substantial [32–35]. During this period, there were increasing trends in both nominal seabird catch rates and fishing effort. The annual nominal seabird catch rate was a mean of 0.005 seabird captures per 1000 hooks (± 0.002 95% CI) from 2004 to 2013, which significantly increased at a rate of 0.001 captures per 1000 hooks per year, based on a linear regression model (p<0.001, R^2^ = 0.88) [13, 18–20, 27–31, 36]. The number of hooks set per year also significantly increased by about 1 million hooks per year from 2004 to 2013, based on a simple linear regression model (p<0.001, R^2^ = 0.84) [18–20, 27–31, 13, 36]. Thus, the annual seabird catch level has been increasing over the past decade due to a combination of significant increasing trends in fishing effort and nominal seabird catch rate.

This study updated and expanded the scope of Gilman et al. [15]. By fitting observer data from the Hawaii longline tuna fishery to a standardized seabird catch rate model, significant explanatory variables were identified, providing information on potential opportunities to reduce the seabird catch rate through changes in fishing methods and gear designs. Identification of variables that were not significant effects on the standardized seabird catch rate also informs management decisions by identifying changes in fishing methods and gear that would not reduce seabird bycatch. The study assessed whether the two regulatory-required alternative suites of seabird bycatch mitigation measures were significant effects in the standardized seabird catch rate model, and whether the two alternative suites of measures had significantly different standardized seabird catch rates. To contribute to identifying additional opportunities to reduce seabird bycatch levels, the study also compared the number of seabird captures and nominal seabird catch rates within and outside of the area where seabird bycatch mitigation methods are required, determined the proportion of caught seabirds that were captured during the haul vs. the set, and determined the mechanism responsible for seabird captures. The study also explored potential causes of the observed increasing trend in nominal seabird catch rate, in part, to identify mitigation opportunities. The expanded knowledge of the efficacy of alternative seabird bycatch mitigation methods is of relevance both locally and globally to manage seabird bycatch in pelagic longline fisheries.

## Methods

### Study Sample–Catch, Gear, Seabird Local Abundance and Environmental Data

The study sample was obtained from the U.S. National Marine Fisheries Service Hawaii longline observer program dataset from deep-sets made by Hawaii-based longline vessels. Sets were determined to be deep-sets based on the captain’s declared set type and ≥ 15 branchlines between floats. Regulations designed to mitigate sea turtle bycatch require deep-setting tuna-targeting vessels to use ≥ 15 branchlines between floats [37]. We excluded records that had not yet been validated and approved at the time of accessing the observer program dataset. We also excluded sets from designated research trips because experimental treatments may have affected fishing methods, gear and catch characteristics.

The study period was from 17 October 2004 to 19 May 2014, spanning 9.6 years. The beginning of the study period was selected based on when observers began to conduct seabird scan counts during both setting and hauling operations. The end of the study period was selected based on the date of the start of the final set of the most current available validated and approved trip.

The study sample comprised the number of seabirds observed caught in 9,719 pelagic longline fishing sets over the study period. Those sets were completed during 1,521 fishing trips undertaken by 154 unique vessels. There were 471 seabirds captured, with 91% either a black-footed (48%) or Laysan albatross (43%). While up to six seabirds were caught in a single set, multiple catches in a set were extremely rare, with 72% of 471 seabirds caught as singletons.

The sample included only: (i) records during which there were observations of one or more albatross present during observer bird scans during the set or during the haul, and/or (ii) one or more seabird of any species was observed captured. Thus, records where no albatrosses were observed attending the vessel during setting and hauling, and no captured seabirds were observed, were excluded from the sample. These records were excluded because the observation of no seabird captures is likely a result of an absence of albatrosses at the fishing grounds when gear was being deployed and retrieved, and not likely a reflection of seabird susceptibility to capture [15, 38]. We only considered the presence or absence of albatross species to determine whether to include a set in the study sample, as reviewed in the Introduction and presented above, as captures of other seabird species are very rare events in this fishery [13, 18–20]. Records were also excluded from the sample used to fit to the standardized catch model due to missing values for one or more of the terms included in the model.

Here seabird ‘captures’ are broadly defined to include observed events of: (i) pre-catch escapements, when seabirds were temporarily caught via hooking or entanglement but escaped prior to being landed onboard; (ii) pre-catch losses, when dead seabirds fell from the gear during hauling and were not retrieved by crew; and (iii) captures, when seabirds were caught in the gear and landed onboard during hauling. While the first two interactions can be defined to be pre-catch losses and not capture events [35, 39], the Hawaii longline observer program data collection protocol includes these pre-catch events as captures.

We modelled total seabird catch as a function of potentially informative covariates and factors. The terms included in the model, and several additional variables that were explored but not included, were considered due to evidence from previous studies that they have potentially significant effects on seabird catch rates in pelagic longline fisheries [2, 8, 15, 17, 38, 40–45]. Some variables were explored because they are relevant to the seabird bycatch mitigation regulations for this fishery [14]. Definitions of the terms that were included in the model are:

- Year: Based on the date of the start of the set.
- Month: Based on the date of the start of the set.
- Two dimensional spatial location by individual year: In latitude and longitude, of the vessel at the start of the set, for each year in the study sample.
- Mean density of albatrosses: The mean number of Laysan and black-footed albatrosses attending the vessel during setting and hauling. During setting and hauling observers estimate the number of individuals of each seabird species within 137 m of the vessel by conducting a visual scan 360 degrees around the vessel from their observation post [46]. Observers make low, medium and high estimates for each scan count. We used the average of the medium category estimates.
- Side vs. stern setting: The vessel side set, with a line shooter, if used, mounted on the side of the vessel ≥ 1 m from the stern corner, and deployed the mainline and branchlines from the side of the vessel [46], or otherwise did not employ these gear setting methods. On 9 April 2007, observers began to separately record whether a vessel set the mainline and branchlines from the side of the vessel, and whether a bird curtain was deployed. Prior to this change, observers had recorded whether or not a set employed all elements of the regulatory-defined suite of measures that includes side setting, reviewed in the Introduction [13, 14]. For sets during this earlier period recorded as not meeting the regulatory definition of a side set, we assumed that the vessel was stern setting and did not deploy a bird curtain, possibly introducing false negatives.
- Blue-dyed vs. untreated bait: Was blue-dyed or untreated bait used during setting. Bait is recorded as blue-dyed when the bait is at least same intensity as a government blue color standard [46].
- MEI: Annual average multivariate El Niño-Southern Oscillation (ENSO) index (MEI) value as a regional-scale ocean-climate covariate [47, 48]. MEI is based on six variables: sea level pressure, zonal and meridional surface wind components, sea surface temperature, surface air temperature and total cloudiness fraction of the sky [47, 48]. MEI values are normalized, where an MEI value of 0 corresponds to the mean MEI value for the reference period of 1950 to 1993. Positive MEI values represent El Niño phase-like conditions, and negative values represent La Niña phase-like conditions [47, 48].

Eight variables were explored but excluded as model terms upon determining that they had no significant effect or added very little to improve model fit. These were: Time of day of initiating setting; Beaufort wind force value; individual and interacting term of branchline weight amount and leader length; offal and spent bait discharge practices during setting and hauling; hook shape and size; lunar illumination [44] by calculating the day of the synodic lunar cycle (moon age or lunation) using the date of the start of the set and applying the celestial mechanics algorithms in Danby [49]; and mean monthly sea surface temperature (SST) for the north central Pacific Ocean region drawn from the Extended Reconstructed SST v4 series [50].

Six additional potentially significant explanatory variables that were excluded as model terms due to data quality constraints, duplication of other terms, or a lack of variability were:

- Regulatory-defined alternative suite of seabird mitigation methods: Records were categorized into those that employed: (i) one of the two alternative regulatory-defined suites, defined in the Introduction; (ii) sets that met both suites; and (iii) sets that did not meet either of the two suites. The term was excluded in part because there were incomplete parameter estimates for the ‘both’ category, likely due to the extremely small sample size (108 sets, zero seabird captures).
- Bait type: All sets used predominantly small fish species for bait, with saury (sanma, Cololabis spp.) used in >80% of records.
- Blue-dyed vs. untreated bait upon hauling: Almost all (98%) records where blue-dyed bait was used during the set also had bait that was determined to still meet the definition of being blue-dyed upon hauling.
- Thawed bait: Almost all (98%) sets using blue-dyed bait also used completely thawed bait.
- Bird curtain: Most (89%) of the sets using side setting also used a bird curtain.
- Other mitigation methods: A small sample size (5% of records) used a towed buoy, tori line or water spray during setting.
- Mainline line shooter: For all but 10 records a line shooter was used to set the mainline. Due to this lack of variability, and also because mainline tension is not likely to affect seabird catch rates in this fishery, the term was excluded. [Line shooter use is unlikely to affect seabird catch rates in this fishery because Laysan and black-footed albatrosses only access baited hooks at or near the surface [8], and because a line shooter would not likely affect the baited hook sink rate until hooks sank to almost the full length of the branchline below the mainline (branchlines were a mean of 12.5 m long in the current study sample) [8, 45, 51].]

### Statistical Modeling Strategy

Zero seabirds were captured in ca. 96% of the 9,719 sets. To address this apparent excess zeros, and perhaps overdispersion, we considered modelling the seabird catch data using either a zero-inflated Poisson or zero-inflated negative binomial likelihood. We also considered a negative binomial likelihood that accounts for overdispersion only as a means to assess the need for a zero-inflated likelihood. The potential nonlinear functional relationship between seabird catch (which is a rare event) and various informative covariates and factors also needed to be explored, as well as possible interactions between covariates and factors. The data were sampled from sets within trips within unique longline fishing vessels so there were three hierarchical levels of random effects that required exploration. Additionally, the longline fishing sets are geo-referenced and we wanted to account for spatial effects. One robust way to explore these four challenges (excess zeros and appropriate model likelihood, nonlinear functional form, multi-level or hierarchical sampling structure, spatial effects) was to use model boosting techniques [52] in conjunction with fitting generalized additive mixed models for location, scale and shape (GAMLSS: Mayr et al., [53]) to the geo-referenced seabird catch data. The GAMLSS approach enables modeling both the response mean and dispersion as a function of potentially informative covariates [53].

We fitted an ensemble of exploratory GAMLSS models with the canonical link functions using the *gamboostLSS* package [54] for the R statistical modeling and graphics language [55]. We included a wide range of potentially informative covariates and factors, such as fishing effort (log offset of the number of hooks per set), 2D spatial effects, the three random effects (vessel, trip, set), and various variables described above. We used penalized spline base-leaners for nonlinear effects, bivariate tensor product P-splines for the spatial base-leaner, ridge-penalized base-leaner for random effects (intercepts only) and ordinary least square base-leaners for all linear or factor effects included in the model ensemble [52]. Variable and model selection was based on k-fold cross-validation and stability selection procedures [56]. Model comparison between likelihoods considered here (negative binomial, zero-inflated Poisson, zero-inflated negative binomial) was based on the predictive risk metric [54]. This exploratory approach for zero-inflated and high dimensional data helped us to identity: (i) the model likelihood, (ii) a reduced set of covariates and factors (and appropriate functional form), and (iii) the random effects that are most appropriate for the next step, involving spatio-temporal modeling of the seabird data using a geoadditive GAMM approach [57, 58] used previously for seabird bycatch investigation [17, 38], and outlined below.

### Statistical Modelling of Seabird Catch Rates

We modelled the seabird catch rates using a generalized additive regression modeling approach with fixed and random or mixed effects, referred to as a generalized additive mixed model (GAMM). This approach allowed for flexible specification of both the error and link functions, enabled arbitrary specification of the functional form for each continuous covariate included in the model, and accounted for the mixed effects due to the hierarchical sampling scheme here of sets, trips and vessels [57]. Model likelihood, functional form and covariate set were informed by results from the boosted GAMLSS approach outlined above. The GAMM with a simple hurdle-form of zero-inflated likelihood [59] and canonical link functions was then fitted using: (i) thin plate regression splines to model nonlinear covariate effects except for the seasonal effect, where a cyclic cubic regression spline was used to reflect cyclical seasonal behavior [58]; (ii) a two-dimensional Duchon-spline surface smoother to account for structured spatial effects attributable to the geospatial location (latitude, longitude: Miller et al. [60]) of each longline set; (iii) a tensor product of a 2D Duchon-spline surface and a time effect with cubic regression spline basis used to account for any spatial trend over the study period [61]; (iv) log offset for the sampling effort (number of hooks per set); and (v) vessel- or trip-specific heterogeneity incorporated as a random effects (random intercepts) term to account for the multilevel sampling structure of the data set. A set-specific random effect would be redundant as the use of ‘set’ here is an observation-level effect that is far better dealt with implicitly using either a zero-inflated Poisson likelihood or explicitly using a zero-inflated negative binomial likelihood [62]. Furthermore, the boosting GAMLSS exploration found no support for a set-specific random effect.

The spatially explicit GAMM constructed here is known as a geoadditive GAMM [63] and was fitted using the *mgcv* package for R with all smoothness parameters estimated using REML [58]. Random effects were implemented using a smoother term with a random effect marginal basis [eg., s(vessel, bs =“re”)]. Tests for inclusion of random effects was based on a method proposed by [64] and implemented in *mgcv*. Adequacy of model fit was assessed using residual diagnostics implemented in *mgcv* [65] as well as percentage of the null deviance explained and review of the parameter coefficients and standard errors [58, 66]. Visualization of the estimated structured 2D-spatial effect over time was done using the vis.gam() function in *mgcv* coupled with the legend functions provided by the *itsadug* package for R [67] and the *maps* and *mapdata* packages for R [68, 69].

### Seabird Haulback Condition, Mechanism of Capture, and Captures during Setting vs. Hauling

The condition (alive, dead or unknown) of each caught seabird upon haulback (at-vessel condition before being handled by crew) was analyzed. The number of seabirds caught by anatomical hooking location, or by entanglement but not also hooked, was also assessed. The number of seabirds observed becoming caught (hooked and/or entangled) while crew members were retrieving gear was determined. Seabirds observed coming up on the gear from the soak (i.e., seabirds that were already hooked and/or entangled in the gear when crew hauled the gear up to the sea surface from depth) and/or were observed to be waterlogged and with rigor mortis was also determined.

### Seabird Captures and Bycatch Mitigation Methods North and South of 23° N. Latitude

The proportion of seabirds observed captured in the areas where regulations require (north of 23° N.) vs. do not require employment of seabird bycatch mitigation methods (south of 23° N.) was determined. The proportion of sets made north and south of 23° N. that employed seabird bycatch mitigation methods required to be used when fishing north of this boundary was also determined.

## Results

### Model Likelihood and Minimal set of Covariates and Factors

Model boosting of the GAMLSS models and the various covariate and factor ensembles suggested that the more appropriate likelihood based on the predictive risk metric was the zero-inflated Poisson with a minimal set of covariates and factors. Model terms for the mean or expected response component were month as a cyclic covariate, albatross density in the vicinity of the vessel as a nonlinear covariate, the 2D spatial effect, MEI as a nonlinear effect and a cofactor of side vs. stern setting. And for the variance component, albatross density was included as a nonlinear covariate. Other covariates, factors and random effects may be important but added very little to improve model fit. The zero-inflated Poisson likelihood model (risk metric = 1676.2) was a better fit to the seabird catch rate data than a model using the zero-inflated negative binomial likelihood (risk metric = 1723.8) or just the negative binomial likelihood (risk metric = 1685.2). Therefore, the seabird catch data displayed significant zero-inflation but not overdispersion.

### Spatio-temporal GAMM

Other than the variance component identified in the best-fit GAMLSS model, we used the same ensemble and likelihood to inform our geoadditive GAMM approach. However, we also included the year effect (as this is of management and conservation interest), blue-dyed bait vs. untreated bait to compare with the side-setting effect (as this is also of management interest) and a random intercepts effect for each of the 154 longline vessel as this is still part of the sampling architecture of the study. We also extended the spatial effect to be a spatio-temporal effect with separate 2D spatial effects for each of the 11 years in the study sample.

Table 1 summarizes the dataset used in the standardized catch rate model through a multi-dimensional contingency table using four of the variables determined to be significant effects in the standardized catch rate model (season, albatross density, side vs. stern setting, blue-dyed vs. untreated bait). Table 1 provides summary statistics of seabird capture rates based on a binomial estimator with Clopper-Pearson confidence intervals [70]. The average seabird nominal catch rate point estimate was higher during first half of year (mean of 0.023), than during the second half of the year (mean of 0.005). Within each season, catch rates trended upwards with higher albatross density and when stern vs. side setting. Categories 7 and 8, fishing in the first half of the year with higher albatross density and stern setting, had the highest seabird catch rates, which were significantly higher than all other categories except for category 13, fishing in the second half of the year with higher albatross density, with side setting and blue-dyed bait (Table 1). The nominal seabird catch rate during the study period (0.021 captures per 1000 hooks) (Table 1) was 74% lower than the rate prior to seabird regulations coming into effect (0.080 captures per 1000 hooks) [15].

**Table 1:** Multi-dimensional contingency table, providing summary statistics of seabird capture rates based on a binomial estimator with Clopper-Pearson confidence intervals [70], Hawaii longline tuna fishery, 2004–2015.

								Catch rate (no. per 1000 hooks)		
Category no.	Season	Albatross density	Side vs. stern set	Blue-dyed vs. untreated bait	No. sets	No. hooks	No. seabird captures	Point estimate	95% CI	
1	Jan-Jun	<1.5	Side-set	Blue bait	86	189,993	0	0.000	0.000	0.019
2				Untreated bait	811	1,966,244	10	0.005	0.002	0.009
3			Stern-set	Blue bait	1282	2,834,718	35	0.012	0.009	0.017
4				Untreated bait	1030	2,225,298	30	0.014	0.009	0.019
5		≥1.5	Side-set	Blue bait	66	156,217	1	0.006	0.000	0.036
6				Untreated bait	1028	2,503,049	59	0.024	0.018	0.030
7			Stern-set	Blue bait	1764	4,060,741	248	0.061	0.054	0.069
8				Untreated bait	338	762,467	46	0.060	0.044	0.081
9	Jul-Dec	<1.5	Side-set	Blue bait	44	105,140	0	0.000	0.000	0.035
10				Untreated bait	643	1,586,850	3	0.002	0.000	0.006
11			Stern-set	Blue bait	1109	2,567,462	11	0.004	0.002	0.008
12				Untreated bait	590	1,297,174	11	0.009	0.004	0.015
13		≥1.5	Side-set	Blue bait	4	9,806	0	0.000	0.000	0.376
14				Untreated bait	298	742,886	5	0.007	0.002	0.016
15			Stern-set	Blue bait	539	1,312,260	10	0.008	0.004	0.014
16				Untreated bait	87	195,335	2	0.010	0.001	0.037

**Fig 1:**
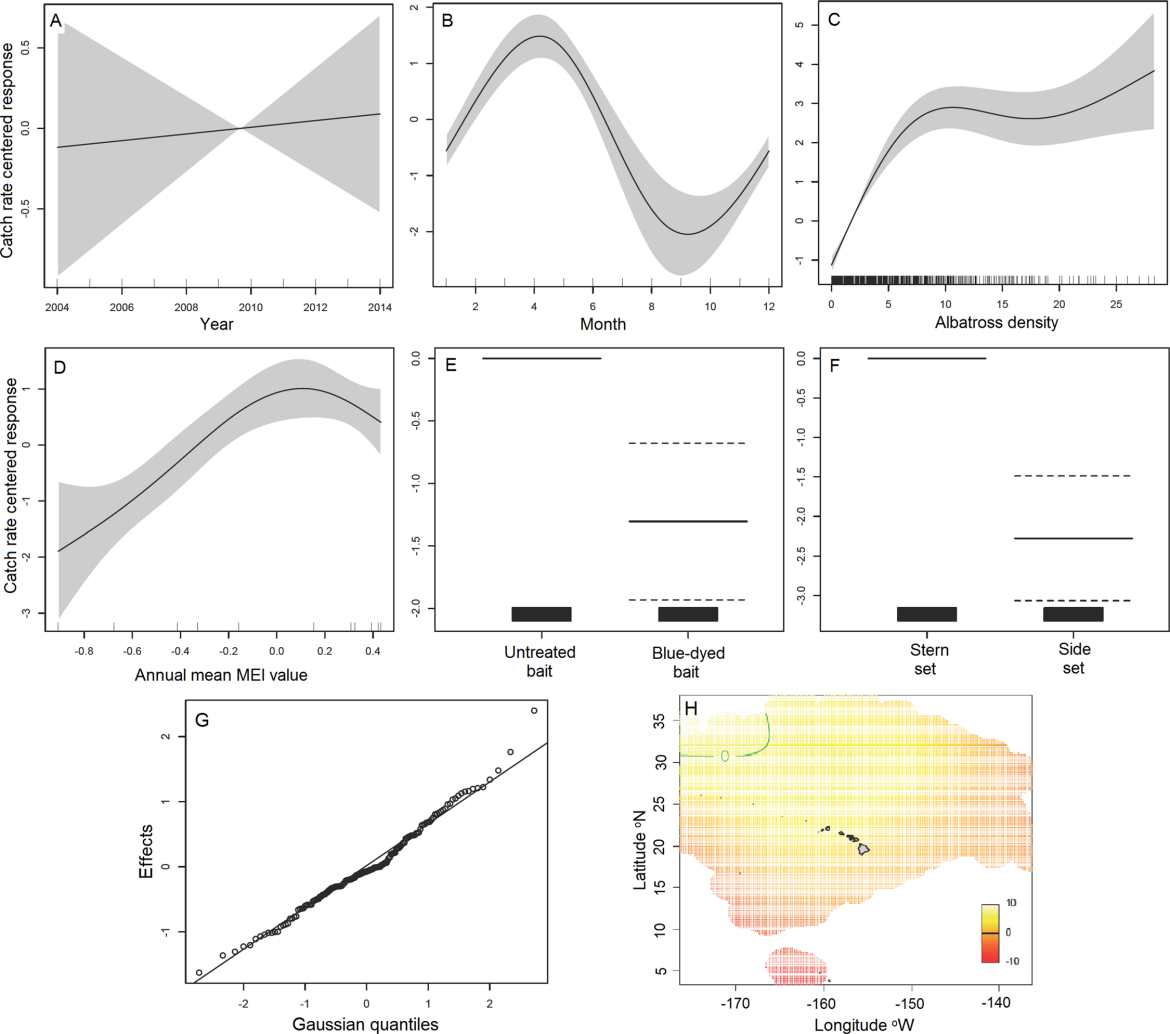
Graphical summary of geoadditive GAMM analysis, Hawaii longline tuna fishery, 2004–2014. The response variable, seabird catch rate, is shown on the y-axis as a centered smoothed function scale to ensure valid pointwise 95% confidence bands. Covariates and factors are shown on the x-axis: (A) year, (B) month, (C) mean albatross density during setting and hauling, (D) annual mean MEI value, (E) untreated vs. blue-dyed bait, (F) side vs. stern setting, (G) quantile plot of random effects for the 154 vessels, and (H) structured spatial effect for all 11 years combined. Color scale units in (H) represent centered scale values. For covariates, solid curves are the smoothing spline fits conditioned on all other covariates and factors, and the shaded areas are bounded by pointwise 95% confidence curves around the fit in each panel. For factors, solid bars are the mean, dashed bars are the 95% confidence interval, and the first factor is the reference level, which is centered at zero on the y-axis. Vertical ticks or rug on the x-axis in each panel show the data distribution.

Table 2 presents a summary of the geoadditive GAMM estimates of parameter coefficients for categorical terms and of the significance of smooth continuous terms and the one random effects term. Fig 1 presents the results of the geoadditive GAMM. All factors, covariates and random effect terms were significant except for the covariate year (Table 2, Fig 1A), a finding consistent with the GAMLSS exploratory results. The spatio-temporal effect was significant with most space x year terms also significant (Table 2). The fitted model explained 82.4% of the null deviance. Residual diagnostics showed no anomalous patterns.

**Table 2:** Summary of geoadditive GAMM fit to seabird catch data, Hawaii longline tuna fishery, 2004–2015. edf = effective degrees of freedom, te() = tensor product, s() = nonparametric smoother. Model terms are defined in the methods section.

**Parametric coefficients**				
	**Estimate**	**SE**	**z-value**	**P**
Intercept	-12.5815	0.3203	-39.281	<0.001
Blue-dyed bait	-1.3056	0.3130	-4.172	<0.001
Side-set	-2.2757	0.3943	-5.772	<0.001
**Approximate significance of smooth terms**				
	**edf**		**Chi sq**	**P**
s(year)	1.000		0.085	0.77
s(month)	1.933		71.520	<0.001
s(albatross_density)	2.924		117.988	<0.001
s(MEI)	2.565		19.917	<0.001
te(lon,lat): year 2004	1.000		3.851	<0.05
te(lon,lat): year 2005	4.830		11.425	0.07
te(lon,lat): year 2006	3.001		7.829	<0.05
te(lon,lat): year 2007	8.429		29.267	<0.001
te(lon,lat): year 2008	7.913		48.864	<0.001
te(lon,lat): year 2009	9.065		32.116	<0.001
te(lon,lat): year 2010	7.542		28.497	<0.001
te(lon,lat): year 2011	8.966		42.372	<0.001
te(lon,lat): year 2012	4.320		10.896	0.06
te(lon,lat): year 2013	3.842		24.523	<0.001
te(lon,lat): year 2014	3.007		2.491	0.48
**Random effects term**				
	**edf**		**Chi sq**	**P**
s(vessel)	58.524		161.530	<0.001

Each panel of the GAMM plot in Fig 1 is on the same y-axis scale, allowing for the identification of the relative contribution of each covariate and factor in explaining model variability. The terms’ relative effect sizes on seabird catch risk, from highest to lowest effect size, were: albatross density, month, annual mean MEI, side setting, blue-dyed bait and year (Fig 1). Fig 1B shows the seasonal effect, with the highest seabird catch rate around April and lowest around September. The catch rate increased steeply as albatross density around the vessel increased to a threshold of about 10 individuals within 137 m of the vessel (Fig 1C). Seabird catch risk increased with increasing mean annual MEI values (Fig 1D). There was significantly reduced risk of seabird capture with the use of blue-dyed vs. untreated bait (Fig 1E) and with side vs. stern setting (Fig 1F). The 95% confidence intervals of blue-dyed bait and side setting in Fig 1E and 1F overlap slightly, but analysis of the parameter estimates from the geoadditive GAMM fit (Table 2) showed that side-setting reduced the risk of capture significantly more (at alpha = 0.05 level) than using blue-dyed bait, although the difference was marginal (P = 0.05). Fig 1G shows a quantile plot of the vessel-specific random effects, which is consistent with the intercepts for vessels being sampled from a Normal distribution, an important assumption of the geoadditive GAMM.

**Fig 2:**
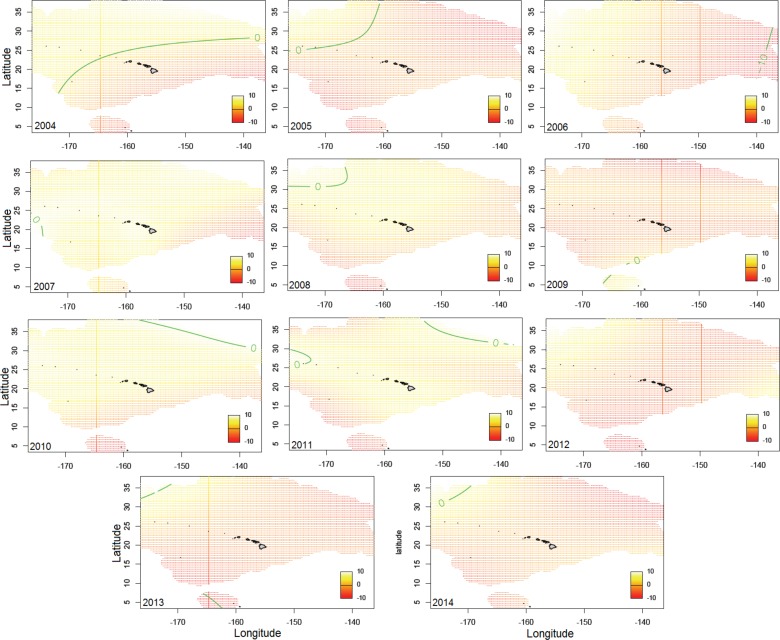
Spatio-temporal effect. Structured spatial effect for each of 11 years (2004–2014), Hawaii longline tuna fishery. Color scale units represent centered scale values as for GAMM response variables shown in Fig 1.

Fig 1H shows the residual overall 2D spatial effect averaged over all 11 years of the study sample. Seabird capture risk was highest to the northwest of the main Hawaiian Islands, with decreasing seabird catch rate eastward and southward. The spatial effect for each of the individual 11 years, after accounting for the other covariates and factors in the geoadditive GAMM, is shown in Fig 2. There was relatively higher seabird capture risk in 2007, 2008 and 2010, and decreasing risk in more recent years throughout the fishing grounds. Seabird catch rates were highest in the northwest in most of the 11 individual calendar years of the study sample (Fig 2), consistent with the spatial effect averaged over the full study period (Fig 1H).

### Haulback Condition, Mechanism of Capture, and Captured During Setting vs. Hauling

Table 3 identifies the haulback condition, number of seabirds observed getting captured while gear was being hauled and their haulback condition, and number of seabirds observed coming up on the gear from the soak, or otherwise waterlogged and with rigor mortis, their haulback condition, and mechanism of capture. Of 471 observed seabird captures, 5% were alive and 95% were dead upon retrieval, before being handled by the crew or observer (Table 3). Observers witnessed 16 seabirds being captured during hauling, all of which were alive. Observers documented 96 dead seabirds on the gear as the gear came up from the soak and/or being waterlogged and with rigor mortis. Almost all caught seabirds were hooked; only 3% were entangled in line and not hooked (Table 3). Of the seabirds captured via hooking, most (83%) ingested the hook with the hook lodged in the beak or mouth (47%) or more deeply in the esophagus or deeper (36%). The remainder was either foul hooked (11%) or the hooking location was not identified (6%) (Table 3).

**Table 3:** Seabird haulback condition, number caught by anatomical location of hook or by entanglement but not also hooked, number observed captured while gear was being retrieved and their haulback condition, and number observed coming up on the gear from the soak and their haulback condition, observer program data, Hawaii deep-set pelagic longline tuna fishery, 17 October 2004 to 19 May 2014.

	Haulback condition		No. captured by				No. observed getting caught while gear was being hauled		No. observed coming up from the soak	
Species/group	No. alive	No. dead	Hooked in mouth or internally	Hooked in body	Hooked location unknown	Entangled not hooked	Alive	Dead	Alive	Dead
**Black-footed albatross**	8	217	177	22	21	5	6	0	0	58
**Laysan albatross**	13	190	162	27	4	10	9	0	0	36
**Shearwater spp.**	0	41	38	2	1	0	0	0	0	1
**Brown booby**	0	1	1	0	0	0	0	0	0	1
**Unidentified**	1	0	0	1	0	0	1	0	0	0
**Total**	22	449	378	52	26	15	16	0	0	96

### Seabird Captures and Bycatch Mitigation Methods North and South of 23° N. Latitude

Of 392 fishing operations with one or more observed seabird capture, 82.4% had sets begin north and 17.6% south of 23° N. Of 471 seabird captures observed in the study sample, 83.9% (395) were caught in fishing operations with sets that began north, and 16% (76) south of 23° N. By species, 77%, 93% and 78% of black-footed albatrosses, Laysan albatrosses and shearwater species were caught north of 23° N., respectively. Of the 9,719 records included in the study sample, 73% (7,114) had sets begin north and 27% (2,605) south of 23° N. Table 4 summarizes the proportion of records included in the study sample where sets began north or south of 23° N. where different seabird bycatch mitigation methods were employed.

**Table 4:** Percent of fishing operations (one operation being a set, soak and haul of the gear) where sets began north or south of 23° N. where different seabird bycatch mitigation methods were employed, observer program data, Hawaii deep-set pelagic longline tuna fishery, 17 October 2004 to 19 May 2014.

Seabird bycatch mitigation method^1^	% of fishing operations	
	North of 23° N	South of 23° N
One or both regulatory mitigation methods	90.7	18.8
Side set	33.2	23.8
≥ 45g branchline weight and ≤1m leader	99.9	98.0
Blue dyed bait	66.9	5.1
Thawed bait	89.0	77.7
‘Strategic' offal or bait discards during set	46.6	18.3
‘Strategic' offal or bait discards during haul	70.8	48.3

^1^ Mitigation methods are defined in the Methods section description of GAMM terms.

## Discussion and Conclusions

### Modeling Expected Seabird Catch

#### Year

Year was not a significant effect in the seabird standardized catch rate model that explicitly accounted for the other covariates and factors. The significant nonlinear effect of year observed by Gilman et al. [17] for the period overlapping with the time series of the current study may have been due to not explicitly accounting for MEI value or other index for ENSO phase or for albatross density at the vessel in their catch rate model.

#### Month

Maximizing effort from August to November, and minimizing effort around April/May would minimize standardized and nominal seabird catch rates in the Hawaii longline tuna fishery. Results were generally consistent with Gilman et al. [15], which found higher standardized and nominal seabird catch rates in the first half of the year than the second half for the period 1994–2007, and consistent with Gilman et al. [17] and Gilman et al. [38] who found similar effects in the Hawaii longline tuna and swordfish fisheries, respectively. The observation of highest standardized catch rates from March to May (Fig 1B) corresponds with periods when breeding Laysan and black-footed albatrosses are rearing chicks. At this stage, they make a mix of short and long foraging trips from their breeding colonies in the Northwestern Hawaiian Islands in order to provide a frequent rate of chick-feeding, foraging in areas that partially overlap the distribution of the Hawaii longline tuna fishery grounds [71, 72]. Months with lowest standardized seabird catch rates fall during the post-breeding season for both Laysan and black-footed albatrosses, and initiation of the breeding season for black-footed albatrosses. During the nonbreeding season, individuals that were breeders the previous season have recovered from the demands of breeding, and forage in areas that overlap less with the fishing grounds of the Hawaii longline tuna fishery relative to the distribution of their foraging grounds during the breeding season [71–73].

#### Albatross density

Findings on the effect of albatross density (local abundance) were consistent with several past studies [17, 33, 38, 74, 75]. Local abundance during setting and hauling affects catch rates due to the effect of animal density on catchability [8, 33]. The local abundance of seabirds can also affect their scavenging behavior, where up to some threshold specific to a seabird species complex, the larger the local seabird abundance, the more intense competitive scavenging behavior and risk of capture will be. However, the effect of albatross density observed here may not occur in longline fisheries in other areas. The effect of hierarchical competitiveness between seabird species and between individual birds, as well as the effect of the presence and local abundance of small deep-diving seabirds that retrieve submerged baited hooks and bring them back to the surface where they become available to larger seabird species with poor diving capabilities may be larger effects and potentially override the effect of albatross relative abundance around vessels on albatross catch rates [8, 76, 77].

There was a significant linear increasing trend in albatrosses attending vessels during the study period, based on fitting the study data on seabird scan counts to a linear regression model (p<0.001, R^2^ = 0.81). This is consistent with observations in the Hawaii longline swordfish fishery [38]. This increasing trend in albatross density attending vessels might partly explain the significant linear increasing trend in nominal seabird catch rate (reviewed in the Introduction).

The increasing trend in number of albatrosses attending vessels might be due, in part, to an increasing trend in absolute abundance of black-footed albatrosses. Based on breeding pair counts at their main colonies, over the study period, Laysan albatross absolute abundance was estimated to be stable, while there was an increasing trend in black-footed albatross absolute abundance [21]. The observed increasing trend in albatrosses attending vessels might also have reflected increased scavenging from fishing vessels due possibly to decreasing availability of natural prey due to declining local abundance of tunas and other subsurface predators, which reduces the availability of prey to pelagic seabirds, discussed below. Or, the observed temporal trend in albatross density around the Hawaii longline tuna vessels may be linked to trends in ocean productivity, discussed below.

#### MEI

Increasing MEI value is indicative of decreasing ocean productivity of the north central Pacific Ocean [78]. The findings here on effect of annual mean MEI value suggest that Laysan and black-footed albatross capture risk increases with decreasing ocean productivity, which has decreased in recent years in the central North Pacific Ocean. There has been a significant -1.1% declining trend in annual median chlorophyll in the north central Pacific Ocean from 1998–2012 [79]. Decreasing ocean productivity may have partly explained the observed increasing trend in Laysan and black-footed albatross density around the fishing vessels (discussed above), which in turn might explain why lower ocean productivity increases capture risk. Perhaps during periods of lower ocean productivity the local abundance of seabird prey resources are lower, resulting in increased numbers of birds scavenging from fishing vessels and also more intense scavenging behavior, increasing the risk of capture.

In the western and central Pacific Ocean, ENSO phases are associated with large scale east–west shifts in the Warm Pool and the highly productive convergence zone between the Warm Pool and cold tongue, altering upwelling intensity and the depth of the thermocline in different regions of the Pacific [80, 81]. This variability in the spatial and temporal occurrence of areas of high ocean productivity and variability in thermocline depth result in variability in the horizontal and vertical distributions and recruitment of pelagic species. This includes prey species of North Pacific albatrosses, and species of subsurface predators (e.g., tunas, dolphins) that drive and cluster prey close to the sea surface making them accessible to shallow-diving seabirds [80, 82–84].

Individual pelagic seabird species, and age classes and sexes of a species, have predictable pelagic habitat preferences for foraging [72, 73, 85, 86]. The distributions and possibly foraging behavior, including scavenging from fishing vessels, of individual species of pelagic seabirds may vary in response to variability in the biomass and distributions of their prey resulting from inter-annual ENSO phases and other natural large scale climate cycles, such as longer-scale Pacific Decadal Oscillation (PDO) phases. For example, during La Niña conditions (low MEI values), Laysan and black-footed albatrosses that are brooding chicks at colonies in the Northwestern Hawaiian Islands traveled farther to reach preferred foraging habitat, including the Transition Zone Chlorophyll Front (TZCF) which is farther north from the breeding colonies than during El Niño conditions (high MEI values) [87].

Findings of the MEI value effect suggest that Laysan and black-footed albatrosses obtain a larger food subsidy from longline fishing vessels during periods of low ocean productivity. This might support more stable population abundance and augment resistance and resilience, with direct effects on the structure, processes and stability of their nesting and foraging communities and indirect effects on interconnected systems [35, 88, 89].

The range of annual average MEI values that occurred during the study period represented relatively weak to moderate strength ENSO phases [48]. With a longer data series, encompassing strong to extreme ENSO phases, larger effects on seabird catch rates than observed here might therefore be apparent.

#### Blue-dyed vs. untreated bait

Findings on blue-dyed bait effect were consistent with a previous assessment of observer data from the Hawaii longline tuna fishery [15]. Blue-dyed bait has been observed to significantly lower seabird catch rates (see review in Gilman and Hall [45]). Blue-dyed bait might be more difficult for seabirds foraging from above to see as the contrast between the blue-dyed bait and seawater is reduced. Factors that determine whether dyed bait will have reduced contrast to the sea surface include bait type, the amount of dye absorbed by the bait, sea color and ambient light levels. Squid soaks up dye better than fish with scales. Completely thawed bait soaks up dye better than frozen and partially thawed bait, and the longer the bait soaks, the more dye it will soak up, until some threshold [40]. Alternatively or possibly additionally, the blue color of the bait may make it unattractive to seabirds perhaps because they might be less likely to recognize it as a prey item [90]. Completely thawed bait sinks faster than frozen bait, reducing the duration they are accessible to scavenging seabirds [7]. Almost all (90%) of the sets recorded as using blue-dyed bait met all of the regulatory defined elements of the suite of measures that includes blue-dyed bait, reviewed in the Introduction.

#### Side vs. stern setting

Consistent with findings from this study, previous studies found side setting with a bird curtain vs. stern setting to significantly reduce seabird catch rates in the Hawaii longline swordfish and tuna fisheries [15, 34]. When baited hooks are set close to the side of the vessel hull, seabirds are unable or unwilling to pursue them, and by the time the stern passes the hooks, they have sunk to a depth where seabirds have more difficulty detecting them or that exceeds their diving depth range [34]. Almost all (89%) of sets recorded as side setting met all of the regulatory defined elements of the suite of measures that includes side setting, defined in the Introduction.

#### Side setting vs. blue-dyed bait

Sets using side setting had a marginally significant lower standardized seabird catch rate than sets using blue-dyed bait. Gilman et al. [15] found no significant difference between standardized seabird catch rates of side setting and blue-dyed bait in the Hawaii longline tuna fishery, but employed a substantially smaller sample size from a shorter time series. Discussed above, almost all sets using blue-dyed bait also used all of the other elements of the regulatory suite of measures that includes blue-dyed bait, and likewise almost all sets using side setting met all of the elements of the regulatory suite that includes side setting. The finding from this expanded study suggests that the seabird catch rate would be reduced by having vessels that opt to use the regulatory suite of measures that includes blue-dyed bait switch to the suite including side setting. Of sets made in the area where use of seabird bycatch mitigation measures is required, twice as many used blue dyed bait than side setting (Table 4), indicating a higher preference for blue-dyed bait. Discussed below, compliance with side setting is likely higher than with blue-dyed bait when an observer is not present, based on observations of practices when fishing in areas where seabird bycatch mitigation methods are not required (Table 4). Both methods’ efficacy rely on crew practices, e.g., whether crew throw baited hooks close to the vessel hull and far forward during side setting, and whether crew completely thaw baits and dye them to the regulatory-required color [8, 15].

#### Geospatial location

Gilman et al. [15, 17] also found highest standardized seabird catch rates to the northwest of the main Hawaiian Islands. Displacing effort from this area would reduce seabird bycatch rates. The higher catch rates in this area may be due to the effect of the number of albatrosses attending vessels, resulting from foraging habitat preferences of Laysan and black-footed albatrosses. Albatross mean density during setting and hauling in sets made within the area west of 165°W and north of 25°N (mean of 4.6 albatrosses within 137 m of the vessel during setting and hauling, ± 0.4 95% CI, N = 524 sets) was significantly higher than outside this area (mean of 1.9 albatrosses, ± 0.05 95% CI, N = 9195).

### Variables Excluded from Standardized Catch Rate Model

The observed lack of effect of hook shape on seabird catch rate may have been due to the low proportion of captures that occur via foul hooking. The finding may also have been due to there being a relatively small difference in minimum widths between the predominant circle, J and tuna hooks used in the fishery, and all of the hook types being small relative to Laysan and black-footed albatrosses’ mouth sizes. Two past studies found wider circle hooks had lower seabird catch rates than narrower J-shaped hooks [91, 92]. Another study found no significant difference between albatross catch rates on a wider circle hook vs. narrower 9/0 J-hook, however, there was a small sample size (18 albatross captures [93]). No studies, however, including this one, have assessed the single factor effect of hook shape or hook minimum width on seabird catch rates [45]. The predominant circle hooks used in the Hawaii longline tuna fishery are wider than predominant tuna and J hooks [17]. The less exposed points of circle hooks relative to J-shaped hooks [94] reduce catch rates by reducing the probability of foul-hooking organisms [1]. Thus, hook shape might have a small effect on seabird catch rates in this fishery given a small proportion of captures are by foul hooking (Table 3). For fish and sea turtles, J-shaped hooks tend to deep hook, while circle hooks with little or no offset, when swallowed, tend to catch in the corner of the mouth [2, 95–98]. If hook shape has the same effect on anatomical hooking location in seabirds, then using circle instead of J-shaped hooks of the same minimum width might reduce the degree of injury and increase the probability of post-release survival of seabirds that are alive upon haulback, but this is a small proportion of seabirds caught in the fishery (Table 3). For some species and sizes, larger hook minimum width reduces catch risk [45, 98]. For species that tend to be caught by ingesting the hook, hook size can affect susceptibility to capture, as the larger the hook, the lower the probability that an organism can fit it in its mouth [99]. However, small differences in hook minimum width likely have no effect on the ability of albatrosses to fit the hooks in their mouths. But larger hooks might be heavier, and thus have a faster sink rate, reducing seabird catch risk. Therefore, in the Hawaii fishery, hook shape likely has little effect on seabird catch rates, circle hooks might reduce degree of injury for the few birds caught alive, while using larger hooks might reduce seabird catch rates due to faster sink rates.

Consistent with findings of Gilman et al. [17], but inconsistent with those of Gilman et al. [15], the time of day of initiating setting was not a significant effect on seabird catch rate. This may be due to the relatively small dispersion in the time of day of starting sets, with a mean of 8:09 am and 16% CV.

We explored including a categorical term of sets that employed (i) only one of the two regulatory options of suites of seabird bycatch mitigation methods, (ii) both suites, or (iii) neither suite. Sets using only one of the two suites had a significantly lower standardized catch rate than sets not employing one of the suites (p<0.01), a similar finding as the GAMM categorical terms (Fig 1E and 1F). There was a significantly lower standardized seabird catch rate with sets employing both combinations of methods versus sets using only one suite (p<0.001), however, there were incomplete parameter estimates for the ‘both’ category, due to the extremely small sample size for this category.

The mean Beaufort wind force value during setting and hauling was also not a significant effect on the seabird catch rate in the current study. The result may reflect a moderate dispersion in wind strength (mean Beaufort wind force value of 3.3, 35.5% CV). This variable was a significant effect on standardized seabird haul catch rate in the Hawaii longline swordfish fishery [38]. Albatrosses have improved agility and scavenging ability with higher wind strength [75, 100, 101].

Not found to be a significant effect in the current study as an interacting term or individually, branchline weight amount and distance from the hook have been observed in previous studies to significantly affect seabird catch rates during setting [15, 42, 77, 102] and hauling [38] in pelagic longline fisheries. There was relatively small dispersion in swivel weight amounts (mean 47.8 g, 16% CV; 83% of sets used 45 g swivels), and moderate dispersion in leader lengths (mean 0.53 m, 49% CV; >99% of leaders were ≤1 m). Branchline weight amount, and the distance the weight is from the hook, affect the sink rate of the baited hook and availability to seabirds (see reviews in Clarke et al. [2] and Gilman and Hall [45]).

‘Strategic’ discards, employed in 39% of sets and 65% of hauls, did not significantly affect the seabird catch rate in the current study. Discharging offal from processed catch, spent bait and dead discards on the opposite side of the vessel from the setting or hauling stations may draw scavenging seabirds’ attention away from where baited hooks are available and reduce catch rates [103]. However, this might be a short-term effect, where vessels that routinely discharge offal and other organic material might consistently have a larger number of seabirds and other scavengers attending the vessel [7]. Vessels that routinely retain offal and other organic material during setting and hauling might reduce the number of seabirds and other organisms attending the vessel, reducing catch rates, relative to vessels that routinely discharge during setting and hauling [7, 104]. Retention might also reduce competitive seabird scavenging behavior and foraging intensity, reducing capture risk [7].

Not found to be a significant effect in this study, there is evidence of a significant effect of lunar phase on catch rates of some pelagic species in pelagic longline fisheries [44, 105–108]. Moon phase affects night-time ambient light levels, which may affect the ability of nocturnal foraging seabirds to detect and locate baited hooks when scavenging from fishing vessels. Because the Hawaii longline tuna fishery makes sets predominantly during daytime, and because the majority of seabird interactions occur during setting, this is likely why variability in light levels during the nighttime had no significant effect on seabird catch rates.

Not a significant effect in this study, SST, one of several dynamic environmental variables frequently used to standardize longline catch rates, has been observed to significantly explain species-specific catch rates in pelagic longline fisheries (reviewed in Gilman and Hall [45]). SST, which tends to be negatively correlated with latitude, is an indicator for species-specific habitat suitability, as pelagic predators caught in pelagic longline fisheries have disparate temperature preferences [109].

### Haulback Condition, Mechanism for Capture, and Captures during Setting vs. Hauling

In the Hawaii longline tuna fishery, few seabirds are alive when retrieved and almost all are caught during setting. Seabirds retrieved alive were likely captured during hauling, while seabirds retrieved dead were likely caught during the set. These findings are consistent with Gilman et al. [38]. Seabirds that came up on the gear from the soak or that were waterlogged and with rigor mortis were assumed to have been caught prior to gear hauling, most likely during setting. Seabirds are not likely captured during the gear soak as hooks soak at depths far below the diving capabilities Laysan and black-footed albatrosses. Most birds are caught by ingesting a hook and getting hooked in the beak, mouth or more deeply.

### Seabird Bycatch North and South of 23° N. Latitude

Standardized seabird catch rates were lower south of 23° N than north of this latitude, and there were no seabird catch rate hotspots south of 23° N (Figs 1G and 2). When including records that were excluded from the study sample, except for records lacking information on latitude at the beginning of the set or number of hooks set, the nominal seabird catch rates north and south of 23° N. were 0.011 and 0.002 seabirds per 1000 hooks, respectively. Thus, the nominal seabird catch rate south of 23° N. was an order of magnitude lower than to the north. In the ca. 50% of observed effort that occurred south of this latitude, only 16% of seabird captures occurred.

While few sets made south of 23° N. met either regulatory-defined combination of seabird bycatch mitigation methods (19%) relative to those made north of this latitude (91%), most of the sets made south of this latitude had ≥ 45g swivels located within 1m of the hook and used thawed bait. In addition, the proportion of sets using side setting was similar to north of 23° N. (Table 4). This suggests that vessels that employ this line weighting design, thaw their bait and side set do so regardless of where they are fishing. Conversely, the low use of blue-dyed bait and ‘strategic’ discards south of 23° N. relative to sets made north of this latitude suggest that vessels that employ these methods when at grounds where they are required to employ seabird bycatch mitigation methods (at least when an observer is present) do not voluntarily use these methods when not required (Table 4). This further suggests that, when observers are not onboard, compliance with side setting and line weighting is likely higher than with the other methods, which is important given that ca. 80% of effort is unobserved in this fishery.

### Observer Data Fields and Data Collection Protocols

If more than one gear design is used on a vessel, then observers record the predominant design [46]. Relying on predominant gear design (swivel weight amount, hook type, bait type, leader length, etc.) creates uncertainty on the gear design of the branchline on which individual seabird catch events occurred. Modifying observer data collection protocols to have observers record the gear design on which individual seabirds, and other species of conservation concern, were captured, would eliminate this source of uncertainty [45].

Adding observer data collection fields would improve data quality to support a more robust assessment of whether seabirds are captured during setting vs. hauling. Observers recorded, in a comment field, the at-vessel disposition of only 24% of the observed 471 captured seabirds. Additional fields could be added for observers to document during the haul whether they: (i) observed a seabird come up on the gear from the soak; (ii) observed a seabird being captured during gear retrieval; or (iii) did not observe whether the seabird was caught during the haul or come up from the gear soak. For field (ii), four codes could be used to indicate whether, during hauling, the seabird was captured: (a) on a branchline still attached to the mainline, (b) on a tended branchline (line that crew have unattached from the mainline and are actively retrieving), (c) on an untended branchline (crew have unattached the line from the mainline and temporarily attached it to the vessel with terminal tackle still in the water), and/or (d) via entanglement in the mainline [38]. Finally, a field could be added for observers to record whether the seabird had rigor mortis and/or was waterlogged. This latter field could be used as an indicator of whether the bird was caught during the set vs. the haul, which would be useful for capture events where the observer was unable to observe whether the bird came up from the gear soak or was caught during hauling.

It is not currently possible to determine if records met all of the elements of the suite of regulatory required seabird bycatch mitigation methods that includes side setting. This is because information is not in the observer program database on whether the regulatory-required location for mounting a mainline line shooter was met [14]. The observer program dataset does not contain information on the distance that a line shooter is mounted from the stern corner [46]. Similarly, it is not currently possible to determine if records met the suite of regulatory required methods that includes blue-dyed bait because information is not in the observer program database on whether the regulatory-required prohibition for discharged fish and spent bait to contain hooks was met, and whether a discarded fish was returned to the sea in accordance with the regulations [46].
